# Social Interactions Sparked by Pictorial Warnings on Cigarette Packs

**DOI:** 10.3390/ijerph121013195

**Published:** 2015-10-21

**Authors:** Marissa G. Hall, Kathryn Peebles, Laura E. Bach, Seth M. Noar, Kurt M. Ribisl, Noel T. Brewer

**Affiliations:** 1 Department of Health Behavior, Gillings School of Global Public Health, University of North Carolina, Rosenau Hall CB7440, Chapel Hill, NC 27599, USA; E-Mails: mghall@unc.edu (M.G.H.); peeblesk@live.unc.edu (K.P.); laura.bach9@gmail.com (L.E.B.); kurt_ribisl@unc.edu (K.M.R.); 2 Lineberger Comprehensive Cancer Center, University of North Carolina, Chapel Hill, NC 27599, USA; E-Mail: noar@email.unc.edu; 3 School of Media and Journalism, University of North Carolina, Carroll Hall, CB 3365, Chapel Hill, NC 27599, USA

**Keywords:** Pictorial warnings, graphic warnings, smoking, health communications, social interactions

## Abstract

The Message Impact Framework suggests that social interactions may offer smokers the opportunity to process pictorial warnings on cigarette packs more deeply. We aimed to describe adult smokers’ social interactions about pictorial cigarette pack warnings in two longitudinal pilot studies. In Pilot Study 1, 30 smokers used cigarette packs with one of nine pictorial warnings for two weeks. In Pilot Study 2, 46 smokers used cigarette packs with one of five pictorial warnings for four weeks. Nearly all smokers (97%/96% in Pilot Study 1/2) talked about the warnings with other people, with the most common people being friends (67%/87%) and spouses/significant others (34%/42%). Pilot Study 2 found that 26% of smokers talked about the warnings with strangers. Discussions about the health effects of smoking and quitting smoking were more frequent during the first week of exposure to pictorial warnings than in the week prior to beginning the study (both *p* < 0.05). Pictorial warnings sparked social interactions about the warnings, the health effects of smoking, and quitting smoking, indicating that pictorial warnings may act as a social intervention reaching beyond the individual. Future research should examine social interactions as a potential mediator of the impact of pictorial warnings on smoking behavior.

## 1. Introduction

Tobacco use is the leading cause of preventable morbidity and mortality worldwide, causing nearly six million deaths each year [1]. A large body of observational and experimental research suggests that pictorial cigarette pack warnings are a promising policy solution for communicating information about the health risks of smoking and increasing quit intentions [2,3,4]. Based on strong evidence of their superiority over text warnings, the World Health Organization Framework Convention on Tobacco Control calls for large pictorial warnings to be placed on cigarette packs [5]. As of 2015, 77 countries and jurisdictions—representing 50% of the world’s population—had implemented policies requiring pictorial warnings [6].

Social interactions are a key mechanism through which health communication campaigns, including pictorial cigarette pack warnings, may exert their effects [7,8,9,10,11]. However, researchers are only beginning to study social interactions in the context of pictorial warnings [12,13]. While definitions of interpersonal communication and social interactions vary [8,14], we define social interactions as an exchange between two or more people, in this case sparked by a health message. This conceptualization extends beyond in-person conversations to include non-verbal interactions (e.g., showing someone the message) and virtual interactions (e.g., posting about the message on social media). In addition to the role of social interactions in transmitting messages, communications research has explored the potential for social interactions to serve as a mediator or amplifier of campaign effects [8,9,15]. Similarly, our Message Impact Framework [3] suggests that social interactions triggered by messages can facilitate change in smoking attitudes and beliefs.

Social interactions may be particularly important in the context of anti-smoking communication campaigns because smoking is a social behavior, heavily influenced by peer and social networks [16,17]. Smokers are more likely to know other smokers, and smoking behaviors often have ripple effects within social networks [18]. Moreover, smoking behavior often occurs in social settings, affording opportunities for conversations to take place. Studies of anti-smoking campaigns have found associations between campaign-related interpersonal communication and both smoking behavior and its predictors [19,20,21,22,23,24,25]. Unlike traditional anti-smoking mass-media campaigns, pictorial cigarette pack warnings appear on smokers’ cigarette packs, heightening exposure to the warning and providing a unique opportunity to spark social interactions during the act of smoking. 

Few studies have examined the role of social interactions about pictorial warnings, and these studies have examined the frequency, rather than the content, of these interactions [12,13]. Moreover, these studies focused on conversations about warnings, rather than other types of interactions (e.g., non-verbal, virtual) [12,13]. A deeper examination of the nature of social interactions around pictorial warnings may shed light on how smokers communicate about these warnings with others in their social network. To that end, we sought to describe the frequency, content, and nature of adult smokers’ social interactions about pictorial cigarette pack warnings in two pilot studies.

## 2. Materials and Methods

### 2.1. Participants

*Pilot Study 1.* From February to March 2013, we recruited 30 smokers, ages 18 or older, who we observed smoking cigarettes in public places in North Carolina, USA, or who received referrals from study participants. We defined current smoking as having smoked at least 100 cigarettes in their lifetime and now smoking every day or some days, and we excluded pregnant women, people who smoke only roll-your-own cigarettes, and cigarillo-only smokers.

*Pilot Study 2.* In June and July 2014, we recruited 48 smokers using the same eligibility criteria as Pilot Study 1. We used a variety of recruitment methods, including newspaper advertisements, flyers, and email lists.

### 2.2. Procedures

**Figure 1:**
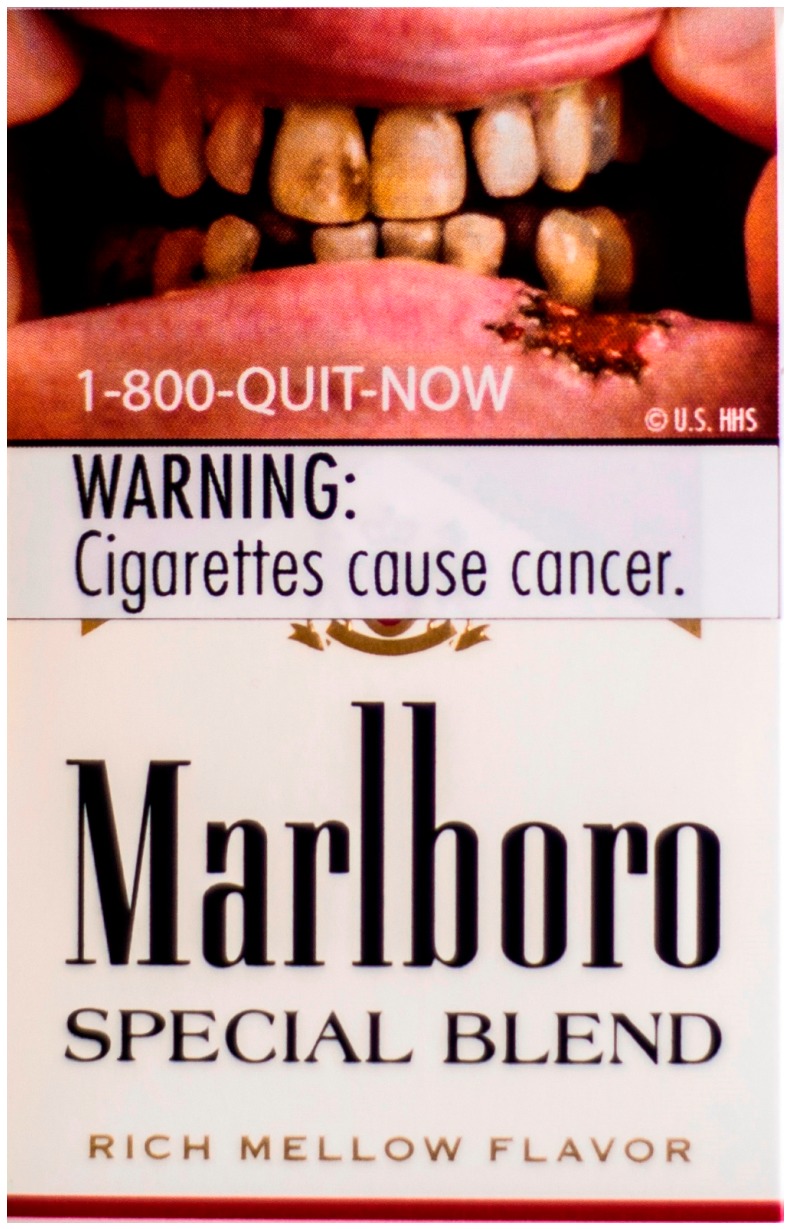
Example of pictorial warning label on pack.

*Pilot Study 1.* A research assistant explained the study to participants and obtained written informed consent. We provided participants with an $80 cash incentive and accompanied them to a nearby store, where we asked them to purchase the amount of cigarette packs they would normally smoke over two weeks as determined in the initial screening. We randomly assigned participants to receive one of nine pictorial warnings that the Food and Drug Administration (FDA) had proposed in 2011 for implementation in the U.S. (Figure 1) (A lawsuit later challenged these warnings, preventing the FDA from implementing them in the U.S. [26].) Then, research staff removed the package cellophane and applied the same pictorial warning labels to the top half of the front and back panels of participants’ cigarette packs, in accordance with the proposed FDA requirements (Figure 1). As applying the labels on the top half of the front panel sealed the flip-top box shut, staff cut through the label to allow the box to open freely. Participants were instructed to smoke cigarettes only from these labeled packs for the duration of the study. Participants took a survey at baseline and then returned to the study offices to complete a follow-up survey two weeks later and received an additional $50 cash incentive. After completing the follow-up visit survey, each participant received information about a local smoking cessation program.

*Pilot Study 2.* Procedures for Pilot Study 2 were similar to Pilot Study 1. After obtaining written informed consent, we randomly assigned participants to receive one of five pictorial warnings, all of which we also used in Pilot Study 1. Participants visited our study offices at baseline and then weekly for four weeks, completing a survey at each visit. Smokers brought eight days’ worth of cigarettes to each of the first four appointments. While participants were taking the survey, study staff labeled their packs using the same procedures as in Pilot Study 1. Participants received a cash incentive at the end of each visit, totaling $185. At the final appointment, each participant received information about a local smoking cessation program. Two of the 48 participants withdrew from the study. The University of North Carolina Institutional Review Board approved the procedures for both studies. Brewer *et al*. [27] provides a more detailed description of the study protocols.

### 2.3. Measures

In Pilot Study 1, the baseline survey assessed demographics and the follow-up survey assessed whether participants talked to anyone about the warnings during the study and with whom they spoke. The survey then included open-ended questions about how conversations about the warning started and the content of those conversations.

The open-ended responses from Pilot Study 1 informed the closed-ended survey items used in Pilot Study 2. Prior to Pilot Study 2, we also refined the survey instrument by conducting cognitive interviews [28] with 15 adult smokers. In Pilot Study 2, participants completed a baseline survey and four follow-up surveys weekly. The surveys asked about the nature of conversations about the warning, including who started the last conversation, conversation partners, topics of conversations, and social media posts about the warnings. Finally, the survey assessed weekly frequency of conversations about the warning, the health effects of smoking, and quitting smoking. 

We defined low income as being at or below 200% of the U.S. federal poverty level. The survey instruments are available upon request.

### 2.4. Data Analysis

We present descriptive statistics for the two studies. For Pilot Study 2 data, we used planned contrasts within a repeated measures analysis of variance (ANOVA) to compare frequencies of social interactions about the warnings (conversations about the warnings, showing someone the warnings) during week 1 to each subsequent follow-up week. We repeated the analyses for social interactions about smoking (harms of smoking, quitting smoking), comparing baseline to each subsequent week. Analyses for Pilot Study 2 excluded two participants who withdrew from the study. Thirteen Pilot Study 2 participants did not receive questions about conversation partners or topics due to a survey malfunction. Analyses used SPSS version 22 and Stata/IC version 13.1. We set critical alpha to 0.05 and used two-tailed statistical tests.

## 3. Results

Participants’ mean age was 30 years in Pilot Study 1 and 43 years in Pilot Study 2 (Table 1). Most smokers were at or below 200% of the US federal poverty level. About 10% of smokers identified as gay or bisexual. The mean number of cigarettes smoked per day was 12 (standard deviation (SD) = 9) in Pilot Study 1 and 11 (SD = 8) in Pilot Study 2.

**Table 1:** Participant characteristics.

Characteristic	Pilot Study 1 (*n* = 30) %	Pilot Study 2 (*n* = 46) %
Age		
18–24 years	40	4
25–39 years	33	37
40–54 years	27	33
55+ years	0	26
Mean (SD)	30 (11)	43 (12)
Female	40	57
Gay or bisexual	10	11
Hispanic	10	13
Race		
Asian	7	4
Black	27	35
White	43	44
Other/Multiracial	23	17
Education		
High school degree or less	33	20
Some college	50	50
College graduate	10	24
Graduate or professional degree	7	7
Low income (≤200% of poverty level)	69	59
Cigarettes smoked per day, mean (SD)	12 (9)	11 (8)

Social interactions about the pictorial warnings were a nearly universal experience in both studies. Twenty-nine of 30 (97%) Pilot Study 1 participants and 44 of 46 (96%) Pilot Study 2 participants talked with others about the warnings at least once. In response to an open-ended question, many participants (60%) in Pilot Study 1 reported that they started conversations about the warning by showing the warning to someone else. Half of Pilot Study 1 smokers (50%) mentioned that conversations began when others noticed and asked about the warning on their pack. Similarly, in Pilot Study 2, when asked how the last conversation about the warning started, 66% of participants reported that they initiated the last conversation about the warning, whereas 34% said that someone else initiated the conversation.

**Figure 2:**
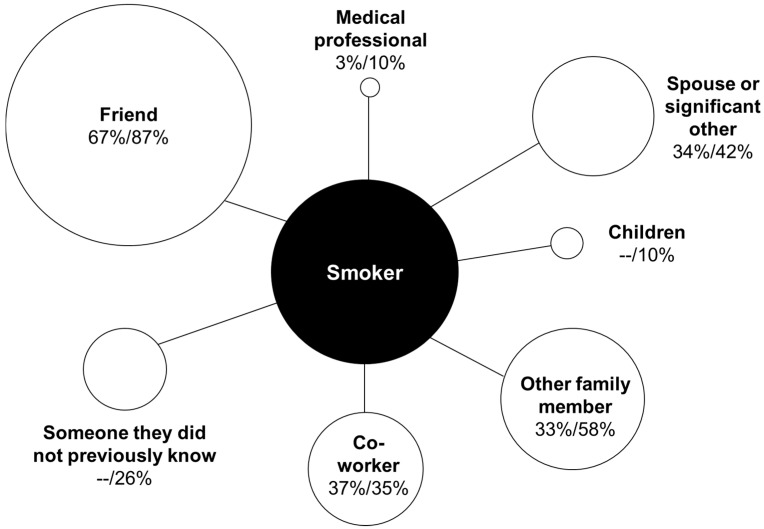
Partners in conversations about warnings, Pilot Study 1/2. Among Pilot Study 1/2 smokers (*n* = 29/31) who had at least one conversation about the warnings. Data were missing for 13 Pilot Study 2 participants; -- not assessed.

Smokers in both studies talked about the warning with a wide variety of people, as shown in Figure 2 (Pilot Study 1 *n* = 29; Pilot Study 2 *n* = 31 after accounting for missing data for 13 people). Most participants reported talking about the warning with a friend (67% in Pilot Study 1 and 87% in Pilot Study 2). Some talked with significant others/spouses, other family members, and co-workers (between 33% and 58% of participants). Few participants talked with a medical professional about the warning (3% in Pilot Study 1 and 10% in Pilot Study 2). Ten percent of Pilot Study 2 participants talked to children, and 26% talked to someone they did not previously know; Pilot Study 1 did not assess these categories. 

The remainder of the findings in our paper reflect data only from Pilot Study 2 participants. Some Pilot Study 2 participants reported talking about the warning with only smokers (17%) or mostly smokers (34%; Table 2). However, conversations with non-smokers were also common, with about half of participants (48%) talking about the warning with a mix of smokers and non-smokers, mostly non-smokers, or only non-smokers. Conversations with non-smokers about the health effects of smoking and quitting smoking were even more common; 70% of smokers reported talking about the health effects of smoking with a mix of smokers and non-smokers, mostly non-smokers, or only non-smokers. Likewise, 63% of participants talked about quitting smoking with a mix, mostly non-smokers, or only non-smokers.

**Table 2:** Conversations sparked by warnings in Pilot Study 2, Week 1.

Conversation Partners	About Warnings n (%)	About Health Effects of Smoking n (%)	About Quitting Smoking n (%)
Only smokers	5 (17)	3 (9)	6 (18)
Mostly smokers	10 (34)	7 (21)	6 (18)
Mix of smokers and non-smokers	11 (38)	17 (52)	15 (45)
Mostly non-smokers	2 (7)	3 (9)	1 (3)
Only non-smokers	1 (3)	3 (9)	5 (15)
n	29	33	33

Notes: Data from people who had at least one conversation about these topics. Data were missing for 13 people in “about warnings” column.

When asked about the content of their conversations, most participants discussed the perceived effectiveness of the warning, including whether the warning would encourage other smokers to quit smoking (77%), would make them want to quit smoking (71%), or would discourage people from starting to smoke (68%; Table 3). Many smokers also discussed the research study (71%) or whether warnings like this should be on all cigarette packs (65%). Few participants reported making fun of the warning (13%). Participants or their conversation partners described the warning as scary (71%), informative/useful (55%), gross (55%), depressing/gloomy (52%), or interesting/engaging (42%; Table 3). Few described the warnings as stupid/pointless (19%) or judgmental/controlling (10%). Two participants reported posting about the warning on Facebook, but no one reported posting on other social media platforms.

**Figure 3:**
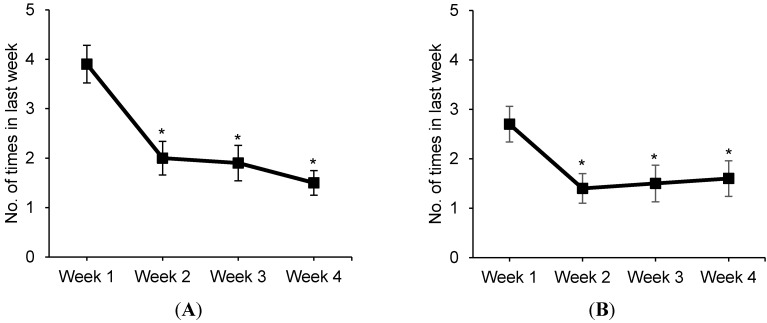
Social interactions about warnings (Pilot Study 2, *n* = 46), * *p* < 0.05, compared to week 1; (**A**) Conversations about the warning; (**B**) Showed someone the warning.

Pilot Study 2 participants had about four conversations about the warning during the first week of the study (Figure 3). Smokers had fewer conversations at weeks 2, 3, and 4 compared to week 1 (all decreases *p* < 0.05), but continued to have almost two conversations per week by the end of the study. Participants showed someone else the warning about three times during the first week of the study, but this number also declined over time (*p* < 0.05). 

**Figure 4:**
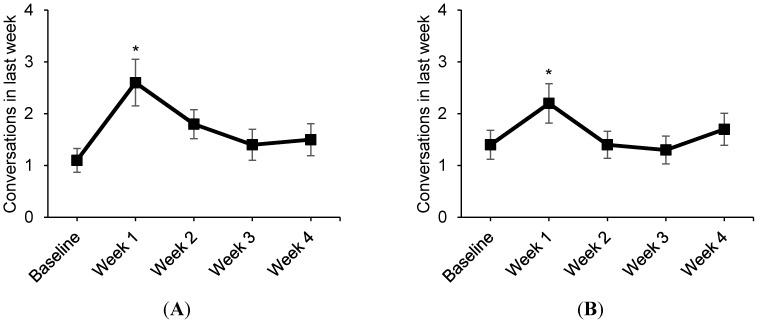
Social interactions about smoking (Pilot Study 2, *n* = 46), * *p* < 0.05, compared to baseline; (**A**) Conversations about health effects of smoking; (**B**) Conversations about quitting smoking.

The number of conversations about the health effects of smoking increased from baseline to week 1 (*p* < 0.05; Figure 4). The number of conversations about quitting smoking also increased from baseline to week 1 (*p* < 0.05; Figure 4). However, the number of conversations about health effects and quitting was not different at weeks 2, 3, and 4 compared to baseline.

**Table 3:** Content of conversations in Pilot Study 2, across all weeks (*n* = 31).

Content of Conversations	n (%)
Topics discussed in conversations	
Whether warning would encourage smokers to quit smoking	24 (77)
Whether warning would make me want to quit smoking	22 (71)
Discussed this research study	22 (71)
Whether warning would discourage people from starting to smoke	21 (68)
Whether warnings like this should be on cigarette packs	20 (65)
Made fun of warning	4 (13)
Descriptions of warnings in conversations	
Scary	22 (71)
Informative, useful	17 (55)
Gross	17 (55)
Depressing, gloomy	16 (52)
Interesting, engaging	13 (42)
Stupid, pointless	6 (19)
Judgmental, controlling	3 (10)

Note: Data from people who had at least one conversation about the warning. Data were missing for 13 people.

## 4. Discussion

Interacting with others about pictorial cigarette pack warnings was a nearly universal experience in both studies, indicating that pictorial warnings exert influence in a fundamentally social manner. Our study found that the pictorial warnings sparked conversations not only about the warnings themselves, but also about the health effects of smoking and about quitting smoking. In other words, pictorial warnings generated meaningful conversations that covered important topics beyond simply talking about the warning itself. We previously reported that most smokers noticed these warnings and about half reported that the warnings caused them to think about the harmful effects of smoking [27]. Moreover, we previously reported that endorsement of positive prototypes of smokers decreased and intention to quit smoking increased between baseline and follow-up. Thus, pictorial warnings may motivate both social interactions and changes in attitudes and intentions. While our study design did not allow us to examine social interactions as a mechanism of change, several theories and frameworks, including the Message Impact Framework [3], highlight social interactions as a possible part of the attitude and behavior change process [3,8,9,10,29]. Two previous observational studies provide further support for the importance of social interactions as a mechanism of attitude and behavior change. A longitudinal study of Australian students ages 7–12 found that frequency of talking about pictorial cigarette pack warnings was associated with lower intentions to smoke, after controlling for individual and parental smoking behavior [13]. Similarly, longitudinal data collected from panels of adult smokers in Canada, Australia, and Mexico found that frequency of talking about pictorial warnings on cigarette packs predicted subsequent quit attempts in adjusted analyses [12]. Our findings, taken together with these previous studies, suggest that social interactions are an integral part of the way that smokers experience pictorial warnings on cigarette packs.

Smokers in our study talked about the warnings with friends, family members, coworkers, medical professionals, and even strangers. Thus, the reach of pictorial cigarette pack warnings extended beyond study participants to others in their social network. Moreover, many participants reported talking with both smokers and non-smokers about the warnings, the health effects of smoking, and quitting smoking. Indirectly influencing non-smokers through health communication campaigns may be a promising way to shift social norms and discourage smoking [30]. For instance, an evaluation of a national anti-smoking campaign in the US, “Tips from Former Smokers,” found that the campaign resulted in millions more non-smokers talking about the dangers of smoking and recommending cessation services to friends or family [30]. Future pictorial warning studies should consider exploring conversations with non-smokers in greater depth, both qualitatively and quantitatively, to determine how these conversations influence subsequent changes in smoking attitudes and behavior. 

The frequency of social interactions peaked at the one-week follow-up visit. This early spike in social interactions may represent a special opportunity for complementary tobacco control campaigns and interventions. However, we found that the frequency of social interactions declined over the four-week period after this initial spike. Given that our study placed only a single warning on smokers’ packs and did not place warnings on the packs of other smokers in a social network, it is possible that the effects of our warnings on social interactions were prematurely attenuated. Previous studies have found that cigarette pack warnings are most effective when newly implemented and that responses to the warnings tend to partially “wear out” over the course of months or years [31,32,33,34]. Studies have found that some smokers actively avoid looking at pictorial warnings [34,35,36] and may become habituated to seeing them over time [34], both of which may contribute to wear-out effects. Thrasher *et al.* (2015) found that the percentage of smokers talking about cigarette pack warnings was relatively high (50%) immediately following the implementation of new pictorial warnings in Canada, but declined at the next survey wave four months later. Several countries, including Australia, Belgium, New Zealand, Mexico, and Trinidad and Tobago, mandate that the content of cigarette pack warnings must change on a regular basis [6]. Research has found that periodic rotation of cigarette pack warnings can help to minimize wear-out effects [32,37]. Indeed, the frequency of talking about cigarette pack warnings has remained relatively stable over time in Mexico, where pictorial warnings rotate every six months [12]. 

Social media may also offer an opportunity to enhance the reach and impact of pictorial warnings [38]. While we found few unprompted interactions about the warnings via social media, campaigns could encourage sharing and interacting about them through social media. In addition to naturally-occurring social media engagement, mass and social media campaigns implemented in conjunction with pictorial warnings may amplify the effects of pictorial warnings [39]. A study of adult smokers in Mexico found that exposure to a complementary mass media campaign was associated with greater attention to pictorial warnings and cognitive elaboration (*i.e.*, the extent to which the warnings made participants think about health risks and about quitting) [40]. Similarly, two studies in Australia found evidence that pictorial warnings and television advertisements worked in a synergistic manner in which the advertisements heightened the effect of pictorial warning exposure on knowledge of the health effects of smoking [41]. Future studies should examine potential synergistic effects of mass media and social media campaigns that complement pictorial warnings.

Strengths of our study include the use of cognitively-tested measures about social interactions, as well as a naturalistic pack labeling protocol that exposes smokers to warnings on their actual cigarette pack [27]. Moreover, the longitudinal study design allowed us to assess trends over time in Pilot Study 2. However, the study took place in the US, where pictorial warnings are not currently on cigarette packs, potentially heightening the immediate novelty of the warnings and thus sparking conversations about the warnings. The external validity of these findings for smokers in other settings and over a longer period of time has yet to be established. The small sample size may have limited our statistical power to detect trends in the frequency of interactions over time.

## 5. Conclusions

Pictorial cigarette pack warnings sparked social interactions about the warnings as well as conversations about the health effects of smoking and quitting smoking. Nearly every participant in our two pilot studies reported talking about the warning, indicating that pictorial warnings function as a social intervention. Future research should explore whether social interactions mediate the relationship between pictorial warning exposure and smoking-related attitudes, intentions, and behavior. Moreover, future studies should examine the content of social interactions and role of conversation partners (e.g., smoker *vs.* non-smoker) as potential moderators of social interactions effects.
